# Microfluidic Formulation of Curcumin-Loaded Multiresponsive Gelatin Nanoparticles for Anticancer Therapy

**DOI:** 10.1021/acsbiomaterials.3c00318

**Published:** 2023-05-04

**Authors:** Yu Xia, Ruicheng Xu, Siyuan Ye, Jiaxuan Yan, Piyush Kumar, Peng Zhang, Xiubo Zhao

**Affiliations:** † School of Pharmacy, Changzhou University, Changzhou 213164, China; ‡ Department of Chemical and Biological Engineering, University of Sheffield, Sheffield S1 3JD, U.K.; § School of Materials Science and Engineering, Changzhou University, Changzhou 213164, China

**Keywords:** microfluidics, nanomedicine, anticancer therapy, targeted drug delivery, gelatin nanoparticles, photothermal ablation

## Abstract

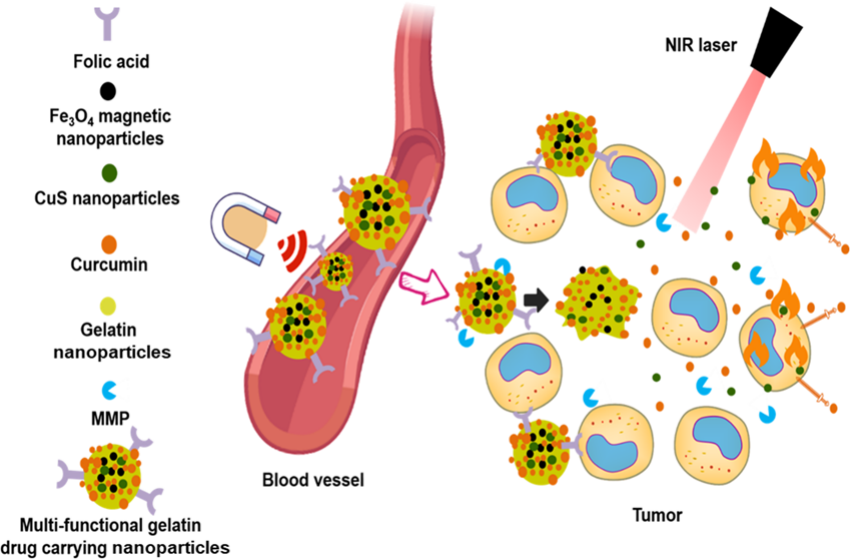

Current anticancer research shows that a combination of multiple treatment methods can greatly improve the killing of tumor cells. Using the latest microfluidic swirl mixer technology, combined with chemotherapy and photothermal-ablation therapy, we developed multiresponsive targeted antitumor nanoparticles (NPs) made of folate-functionalized gelatin NPs under 200 nm in size and with encapsulated CuS NPs, Fe_3_O_4_ NPs, and curcumin (Cur). By exploring gelatin’s structure, adjusting its concentration and pH, and fine-tuning the fluid dynamics in the microfluidic device, the best preparation conditions were obtained for gelatin NPs with an average particle size of 90 ± 7 nm. The comparative targeting of the drug delivery system (DDS) was demonstrated on lung adenocarcinoma A549 cells (low level of folate receptors) and breast adenocarcinoma MCF-7 cells (high level of folate receptors). Folic acid helps achieve targeting and accurate delivery of NPs to the MCF-7 tumor cells. The synergistic photothermal ablation and curcumin’s anticancer activity are achieved through infrared light irradiation (980 nm), while Fe_3_O_4_ is guided with an external magnetic field to target gelatin NPs and accelerate the uptake of drugs, thus efficiently killing tumor cells. The method described in this work is simple, easy to repeat, and has great potential to be scaled up for industrial production and subsequent clinical use.

## 1 Introduction

Cancer is a major disease that seriously affects human life and health.^1−3^ At present, cancer treatment includes surgical resection, radiotherapy, and chemotherapy,^4^ and in recent years, gene therapy,^5,6^ cell therapy,^7,8^ photodynamic therapy,^9−11^ and photothermal-ablation therapy.^12−14^ Each method has its own pros and cons, and anticancer approaches combining two or more methodologies have been shown to be much more effective.^15,16^ Therefore, therapeutic formulations combining multiple treatment methods are more promising for the future of anticancer therapies.

In the past three decades, the development of nanotechnology and its application in medicine (nanomedicine) has evolved rapidly. Nanodrug delivery systems (DDSs) have shown strong anticancer effects due to their ability to prevent the degradation of active pharmaceutical ingredients (APIs), being targeted, controlled release, high bioavailability, low toxicity, and side effects.^17−21^ However, preparing such DDSs is challenging. It is of far-reaching significance to design nanoformulations with stable structures and controllable nanoparticle (NP) sizes.^22^ NPs with sizes less than 200 nm are easier to reach the tumor site due to the enhanced permeability and retention (EPR) effect. Small NPs also have the advantage of being able to easily enter cells through endocytosis. On the other hand, the controlled release of anticancer drugs can be achieved through the fine-tuning of the chemical properties of constituent materials used in NP fabrication. At present, commonly used biomaterials for nanodrug formulations include lipids (e.g., liposomes, lipid nanoparticles),^23−25^ polymers (e.g., PLGA, PLA, chitosan, alginate),^26^ and proteins (e.g., collagen, gelatin, silk, albumin).^21,27−30^ Gelatin is a natural protein that can be obtained by partial acid or alkali hydrolysis, thermal degradation, or enzymatic degradation of structural animal collagen.^31^ Compared with collagen, gelatin does not express antigenicity *in vivo* and is easy to dissolve in an aqueous solution.^14^ Gelatin has become a highly versatile platform for extensive applications in the biomedical field due to its good biocompatibility, biodegradability, nontoxicity, non-antigenicity, and structural plasticity.

Stimulation-responsive nano-DDSs perform significantly better than conventional DDSs and can respond to specific stimuli at the disease/tumor site, leading to changes in their physical and chemical properties and decomposition or fragmentation.^32^ Copper sulfide (CuS) is an inorganic compound, and its NPs have excellent optical and thermal properties. The therapeutic principle behind using CuS NPs is that they can have adjustable absorption peaks in the near-infrared region (700–1100 nm), within which the normal human tissues do not have any absorption peaks, and thus causing no background interference. CuS NPs can increase the temperature in the tumor microenvironment by converting the absorbed infrared light into heat, thereby killing cells. However, because of the complexity of *in vivo* clinical treatment, it is difficult to achieve precisely targeted treatment with only a single NP species, as it is easy for the CuS NPs to flow through the entire body with blood.^33^ Therefore, we have added superparamagnetic iron oxide (Fe_3_O_4_) NPs, which can be easily guided by an external magnetic field. In addition, based on the magnetocaloric function of magnetic NPs (MNPs), thermally responsive drug release can also be controlled by the controlled fluctuation of the applied external magnetic field.

Microfluidics can provide precise control and rapid mixing and has shown great potential in developing new NPs with controllable sizes and compositions.^4,34,35^ The commonly used microfluidic technologies include the T-junction, hydrodynamic flow focusing, staggered herringbone, toroidal mixer, multi-inlet vortex mixers, etc.^34,36^ Recently, a newly developed swirl mixer has shown great potential for the fabrication of lipid NPs and polymer NPs at a large scale with high uniformity.^24^ It has been used for the formulation of silk NPs,^29,37^ ZIF-8 NPs,^35^ curcumin,^38^ doxorubicin,^39^ and Ansamitocin P-3^25^ loaded liposomes for anticancer therapy. The swirl mixer can achieve a high flow rate of 320 mL/min with a mixing time of 0.1 ms, achieving a PDI of 0.06 ± 0.02 for liposome formulation,^38^ demonstrating its great potential for industrial application of nanoformulation.

In this work, the swirl mixer was used to prepare the gelatin NPs with excellent stability for over 2 weeks. Gelatin was functionalized with folic acid (for targeting) and dissolved in DI-water as the aqueous phase, and ethanol or ethanol with Tween-20 was used as the organic phase (antisolvents). CuS and MNPs were blended into the aqueous phase, and curcumin was blended into the organic phase for the fabrication of drug/CuS/MNP-loaded gelatin NPs. We studied the effects of gelatin concentration, solution pH, the composition of antisolvent, the flow rate ratio (FRR), and the total flow rate (TFR) to explore the conditions for controlling particle size and polydispersity. The optimum size was around 90 nm with a PDI <0.1. The curcumin/CuS/MNP-loaded gelatin NPs showed great response to the near-infrared light and magnetic field. The MNPs facilitated cellular uptake of the gelatin NPs, and folic acid enhanced the cancer cell-targeting effect. CuS inside the gelatin NPs enhanced its anticancer effect through near-infrared light irradiation. Overall, the current work demonstrated a promising strategy for the microfluidic fabrication of multicomponent-loaded gelatin NPs for anticancer therapy.

## 2 Experimental Section

### 2.1 Materials

Gelatin type B was obtained from Aladdin Chemistry Co. Ltd. (Shanghai). Carbonate–bicarbonate (CB) buffer and D_2_O were obtained from Beijing InnoChem Science & Technology Co. Ltd. CuCl_2_·2H_2_O, poly(vinylpyrrolidone), Na_2_S·9H_2_O, sodium citrate (C_6_H_5_Na_3_O_7_), folic acid, *N*-hydroxysuccinimide (NHS), 1-ethyl-3-(3-dimethylaminopropyl)carbodiimide (EDC), 1-(3-dimethylaminopropyl)-3-ethylcarbodiimide hydrochloride, dimethyl sulfoxide (DMSO), 4% paraformaldehyde fixing solution, 0.25% trypsin, and glutaraldehyde were purchased from Sinopharm Chemical Reagent Co., Ltd. Dulbecco’s modified Eagle’s medium (DMEM), fetal bovine serum (FBS), and penicillin/streptomycin were obtained from Gibco (Thermos Fisher Scientific). 3-(4,5-Dimethylthiazol-2-yl)-2,5-diphenylte-trazolium bromide (MTT), 4′,6-diamidine-2′-phenylindole dihydro-chloride (DAPI), 2′-(4-ethoxyphenyl)-5-(4-methyl-1-piperazinyl)-2,5′-bi-1*H*-benzimidazole, trihydrochloride (Hoechst 33342), and propidium iodide (PI) were purchased from Beijing Solarbio Bio-Tech Co. Ltd. (China). All other reagents used in this research were of analytical grade.

### 2.2 Preparation of CuS Nanoparticles

A total of 8.525 mg of CuCl_2_·2H_2_O and 20 mg of trisodium citrate were dissolved in 45 mL of deionized water and stirred at 800 rpm for 5 min. The temperature was adjusted to 90 °C before 5 mL of Na_2_S (2 mg/mL) solution was added and stirred for another 15 min to obtain dark green CuS NPs. The reaction was then stopped, and the solution was cooled down to room temperature. The larger particles were removed with a 0.22 μm filter.

### 2.3 Preparation of Fe_3_O_4_ Nanoparticles

The mature oleic acid preparation method was used to prepare Fe_3_O_4_ NPs.^39−41^ Briefly, 2.824 g of iron acetylacetonate, 6.4 g of cetylamine, 6.4 mL of oleic acid, and 60 mL of diphenyl ether were placed in a 150 mL three-port flask, slowly heated to 260 °C, and stabilized for 10 min under constant stirring. The reaction was refluxed for 2 h, after which the reaction was stopped and cooled down to room temperature before 120 mL of absolute ethanol was added and sonicated for 15 min. The solution was then centrifuged at 12,000 rpm for 20 min to remove the supernatant. The pellet was dispersed in 40 mL hexane with 0.4 mL of oleic acid and kept in a 4 °C refrigerator.

### 2.4 Gelatin Modification

The grafting method developed by Zhang et al.^42^ was optimized before use. Briefly, 120 mg of folic acid (FA), 31.2 mg of NHS, and 42 mg of EDC were added to 15 mL of DMSO solution, stirred, and reacted at room temperature overnight to fully activate folic acid. The activated folic acid DMSO solution was added dropwise to 75 mL of deionized water and stirred rapidly, followed by centrifugation at 8500 rpm for 10 min. The supernatant was discarded, and the pellet was washed with water twice before being redissolved with 20 mL of DMSO. Then, 180 mg of gelatin type B was dissolved in 36 mL of CB buffer and heated to 45 °C for 15 min to melt the gelatin. Afterwards, 20 mL of FA–DMSO solution was added dropwise, until the gelatin solution became clear and transparent, and stirred overnight. The reaction product was placed in a 3500 kDa dialysis bag and dialyzed in a 20% DMSO aqueous solution for 48 h before being lyophilized. The folate content was determined according to the literature.^43^ The absorption coefficient (ε) of folic acid is 6200 M^–1^ cm^–1^ at 363 nm. The folic acid content of the modified gelatin was calculated using the equation *A* = ε*BC*, where *A* is the absorbance, *B* is the thickness of the light-absorbing pass length, and *C* is the concentration of the light-absorbing substance.

### 2.5 Microfluidic Production of Gelatin Nanoparticles

Modified gelatin was added to water and heated to 45 °C for 30 min to dissolve completely. Gelatin solutions with different concentrations and pHs were used as the aqueous phase. For the organic phase, 100% EtOH, 95% EtOH, and 95% EtOH mixed with 2% Tween-20 were used as antisolvents. The flow rate ratios between the aqueous phase and the organic phase were adjusted to 1:1, 1:5, 1:10, 1:20, and 1:30, respectively. The total flow rates of the two phases were set at 10.5 and 40 mL/min. The two phases were injected into the inlets of the microfluidic device^24,25,29,44^ for the preparation of gelatin NPs. The particle size and PDI of the gelatin NPs were recorded to optimize the experimental conditions.

### 2.6 Microfluidic Preparation of Curcumin-Loaded Multiresponsive Gelatin NPs

Modified gelatin (100 mg), CuS solution (5 mL of 0.1 mg/mL), and Fe_3_O_4_ (50 μL of 2 mg/mL) were added in a round-bottom flask and heated in a 40 °C water bath for 30 min. The pH of the solution was then adjusted to 11 for use as the aqueous phase. Curcumin was dissolved in a 2% Tween-20–95% EtOH solution at a concentration of 0.5 mg/mL for use as the organic phase. The flow rates of the aqueous and organic phases were kept constant at 0.5 and 10 mL/min, respectively, while being injected into the microfluidic device using a syringe pump (Fusion 4000, Chem XY Inc.). The colloidal solution collected at the end of the device was folic acid-modified gelatin NPs encapsulating CuS, Fe_3_O_4_, and curcumin (Fe_3_O_4_/CuS/Cur@GNPs-FA). Afterwards, glutaraldehyde solution (2%) was added for cross-linking the gelatin. The NP solution was shaken for 30 min before being filtered with a 0.22 μm filter and dialyzed with a 10 kDa dialysis bag for 48 h before being stored at 4 °C for future use.

### 2.7 Drug Encapsulation Efficiency (EE) and Loading Capacity (LC)

The encapsulation efficiency and loading capacity of the drug were determined by centrifugation. Briefly, 2 mL of the prepared drug-loaded gelatin NP solution was centrifuged at 12,000 rpm for 1 h, and the supernatant was discarded. Then, 1 mL of concentrated hydrochloric acid was added to the precipitate and ultrasonicated for 2 h. The solution was diluted to 10 mL with pure water, and the absorbance value was measured with an ultraviolet spectrophotometer (TU-1901PC, Shanghai, China) at a wavelength of 425 nm. The mass *m*_1_ of the drug was calculated according to the standard curve. Then, 1 mL of concentrated hydrochloric acid was added to another 2 mL of the solution from the same sample for demulsification. The absorbance value was measured with the same method after dilution to 10 mL. The mass *m*_2_ of the drug was calculated, and the encapsulation efficiency of the drug-loaded NPs was calculated using eq 1. The precipitate (from 2 mL of solution) after centrifugation was dried overnight at 25 °C, and the mass of precipitate *m*_3_ was weighed. The drug loading was calculated according to eq 2.
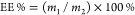
1
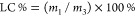
2

### 2.8 Particle Characterization

Fourier transform infrared (FTIR) spectra of folic acid-grafted gelatin lyophilized powders were recorded using grazing incidence attenuated total reflection Fourier transform infrared (ATR-FTIR) spectroscopy (Nicolet iS 10) in the range of 500–4000 cm^–1^. The particle size, polydispersity indexes (PDI), and zeta potentials of different formulations were measured by dynamic light scattering (Nano-ZS90, Malvern Zetasizer, U.K.). The morphologies of the NPs were observed through transmission electron microscopy (TEM).

### 2.9 Drug Release and *In Vitro* Stability

The dynamic dialysis method was used to simulate the release of curcumin from gelatin NPs *in vitro*. Briefly, 2 mL of Fe_3_O_4_/CuS/Cur@GNPs-FA was placed into dialysis bags (MWCO = 10 kDa). The dialysis bags were incubated in 20 mL of prewarmed PBS solution containing 1.5% Tween-80 and 20% anhydrous EtOH (pH = 5.5 and 7.4) with gentle shaking (120 rpm) at 37 °C. The suitability of this *in vitro*-release media for Cur has been reported previously.^45,46^ At specific time points, 2 mL of the released medium was collected and replaced with the same volume of fresh medium. The cumulative amounts of Cur in the 2 mL of samples were determined by ultraviolet (UV) spectrophotometry (TU-1901PC, Shanghai, China) at wavelengths of 425 nm, and their cumulative release was calculated. The test was repeated 3 times, and all data were expressed as the mean ± standard deviation.

The stability of NPs was evaluated in PBS (pH 7.4), NS (stroke-physiological saline solution, 0.9% NaCl), or DMEM medium. In brief, Fe_3_O_4_/CuS/Cur@GNPs-FA was diluted in the medium (1:10, v/v) and stored at 4 °C for 2, 4, 6, 8, 10, 12, 14, and 16 days. Afterwards, the mean size and PDI were measured at different time points.

### 2.10 Cell Cultures

HFF-1 (Human foreskin fibroblasts), A549 (Human lung adenocarcinoma cells), and MCF-7 (human breast cancer cells) were purchased from the China Center for Type Culture Collection (Shanghai, China). The cells were cultured in DMEM and added with 10% FBS and 1% penicillin/streptomycin at 37 °C and 5% CO_2_ in an incubator.

### 2.11 *In Vitro* Cytotoxicity Assays

MTT assay was performed on HFF-1, MCF-7, and A549 cells to rule out any NP-induced cytotoxicity. Briefly, the cells were evenly seeded in a 96-well culture plate at a density of 5.0 × 10^3^ cells per well and cultured at 37 °C and 5% CO_2_ for 24 h. Subsequently, the cells were treated with free-Cur, Fe_3_O_4_/CuS/Cur@GNPs, and Fe_3_O_4_/CuS/Cur@GNPs-FA with different concentrations of Cur (10–100 μg/mL) for 24 h. Different concentrations of blank gelatin, CuS, and Fe_3_O_4_ were incubated with MCF-7 cells to examine biocompatibility. In addition, to evaluate the photothermal effect of CuS, 980 nm near-infrared (NIR) irradiation at 1.0 W/cm^2^ was applied for 5 min. After incubation, MTT solution (10 μL, 5 mg/mL) was added to each well and incubated for another 4 h at 37 °C. Then, the medium was removed and washed with PBS before the addition of 150 μL of DMSO. The absorbance of the solution was measured at 570 nm using a microplate reader (Varioskan LUX, Thermo Fisher) to calculate the percent cell viability. Each treatment was repeated six times in 96-well plates.

### 2.12 Live/Dead Assay

The live/dead cell staining was used for qualitative analysis of cytotoxicity. MCF-7 cells were seeded on cell culture plates at a density of 5.0 × 10^5^ cells/well and cultured for 24 h. The MCF-7 cells were incubated with curcumin, with its final concentration controlled at 10 μg/mL (*n* = 4). In the NIR (+) group, all cells were irradiated with NIR (1.0 W/cm^2^, 5 min) at a wavelength of 980 nm after the cells were cultured with curcumin for 24 h. Cells were washed twice with PBS, followed by incubation with Hoechst 33,342 (2 μg/mL) at 37 °C for 10 min and PI (5 μg/mL) for 20 min. The cell morphology was then observed under a fluorescence microscope (Nikon, Japan, Ti2-U). Blue and red stained cells represent live and dead cells, respectively.

### 2.13 Cellular Uptake

Curcumin itself has green fluorescence, but the fluorescence is quenched quickly under the fluorescence microscope. In order to assess the cellular uptake, a less toxic dye with green fluorescence, coumarin C6, was used instead of curcumin.^47^ MCF-7 cells were cultured for 24 h, and then coumarin C6, Fe_3_O_4_/CuS/C6@GNPs, and Fe_3_O_4_/CuS/C6@GNPs-FA were added. The final C6 concentration was controlled at 10 μg/mL, with 4 repeated wells per group. The cells were incubated in an incubator for 2, 4, 8, and 12 h, respectively. Next, the cells were washed, fixed with 4% paraformaldehyde for 10 min, and stained with DAPI (0.1 μg/mL) for 8 min, followed by imaging using a fluorescence microscope. The same method was used to analyze the uptake of Fe_3_O_4_/CuS/C6@GNPs and Fe_3_O_4_/CuS/C6@GNPs-FA with and without magnetization, respectively.

### 2.14 Flow Cytometry

Flow cytometry was used for the quantitative determination of fluorescent drug uptake. MCF-7 cells were seeded on a 6-well plate at a density of 1 × 10^6^ cells/mL and cultured overnight for 24 h. Coumarin C6, Fe_3_O_4_/CuS/C6@GNPs, and Fe_3_O_4_/CuS/C6@GNPs-FA were added to the wells, respectively, and the concentration of coumarin C6 was controlled at 1 μg/mL. Three repeats were set up in each group, and DMEM medium (containing cells) was used as the control group. After incubation for 2, 4, 8, and 12 h, cells were washed three times with PBS (pH 7.4) to remove unbound drug and harvested separately by trypsin digestion. The internalization efficiencies of the cells were analyzed using flow cytometry (BD Biosciences, Accuri C5). Data from a total of 10,000 events were acquired for each sample, and the mean fluorescence intensity of C6 in the cells was analyzed using FlowJo software. The same method was used to analyze the uptake of Fe_3_O_4_/CuS/C6@GNPs and Fe_3_O_4_/CuS/C6@GNPs-FA with and without magnetization, respectively.

### 2.15 Statistical Analysis

All values in this study were expressed as means ± SD (standard deviation). Differences among groups were analyzed using one-way analysis of variance (ANOVA) with GraphPad Prism 9.0 software. Statistical differences were considered significant at ****P < 0.0001; ***P < 0.001; **P < 0.01; and *P < 0.05.

## 3 Results and Discussion

### 3.1 Characterization of Modified Gelatin

EDC and NHS were used as coupling agents for gelatin and folic acid.^48^ Notably, folic acid has two carboxylic groups denoted as α and γ, and it has been recognized that γ is more reactive than α carboxyl.^49,50^ When the amount of folic acid is small, most of the γ carboxyl groups involve in grafting and react with the primary amine groups in gelatin to form amide bonds. The infrared spectrum of free folic acid has three characteristic peaks at 1601, 1687, and 1487 cm^–1^. Figure S1a shows the FTIR spectrum of the lyophilized powder of folic acid-grafted gelatin recorded in the range of 500–4000 cm^–1^. A distinct characteristic peak between 1650 and 1690 cm^–1^ confirmed the formation of an amide bond between gelatin and folic acid.^51^

There are many characteristic peaks in the hydrogen spectrum of gelatin type B; for example, the methyl resonance of leucine, valine, and isoleucine corresponds to 0.76 ppm, and that of aspartic acid corresponds to 2.84 ppm.^52^ After lyophilizing, 10 mg of folic acid gelatin powder was added to 60 μL of deuterium oxide (D_2_O), and the ^1^H NMR spectrum was measured after heating to 45 °C for 10 min. Figure S1b shows two additional peaks at 1.79 and 2.57 ppm for folic acid-grafted gelatin due to the grafting of folic acid, while the shoulder peak at 7.16 ppm was a characteristic peak due to the aromatic proton resonance of gelatin type B and folic acid.^51^ The above results indicate that folic acid was successfully grafted on gelatin. The folate content was determined according to the literature^43^ and was found to be 56.3 ± 1.4% in the modified gelatin.

### 3.2 Characterization of CuS Nanoparticles

**Figure 1:**
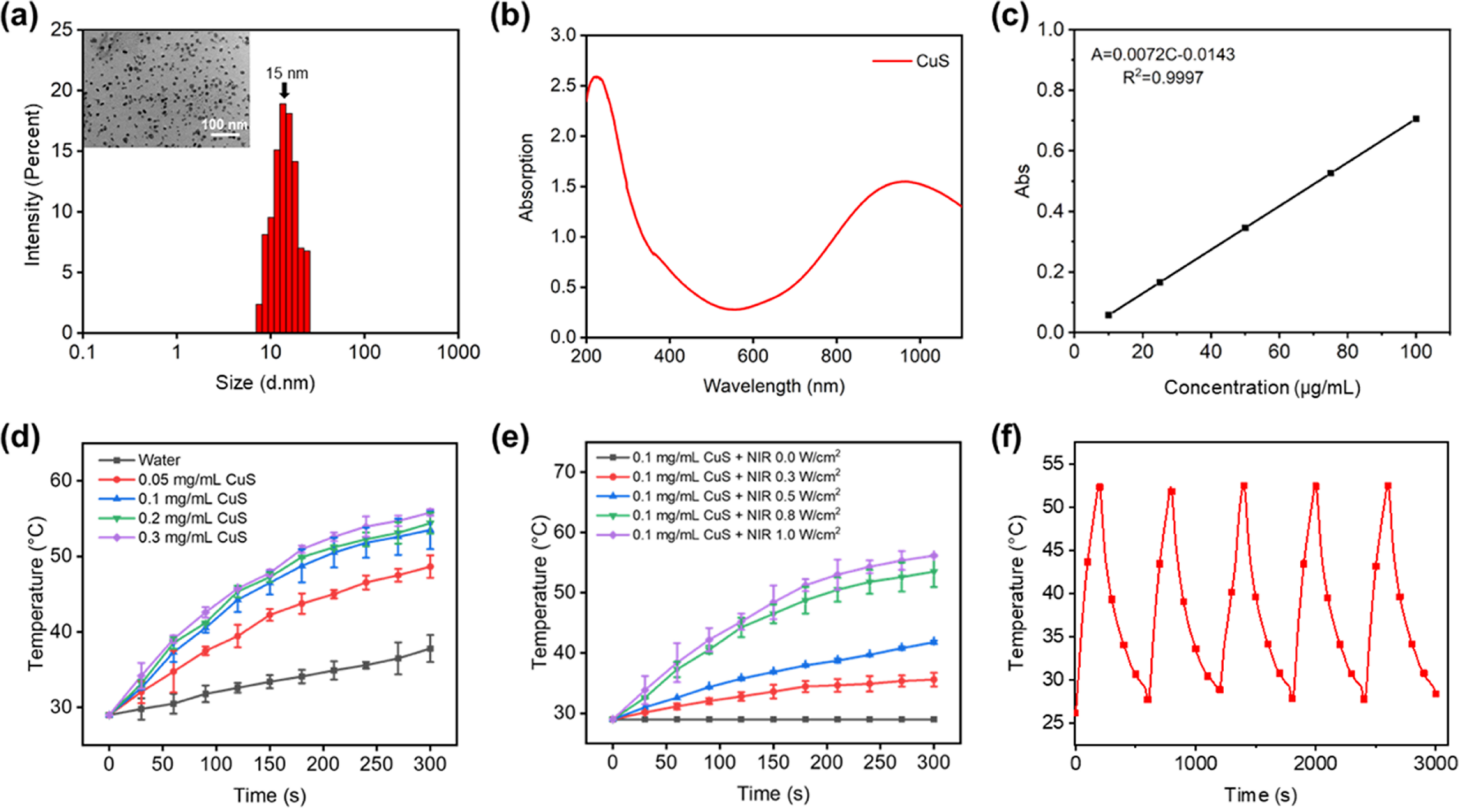
Characterization of CuS nanoparticles. (a) TEM image of CuS with size distribution. (b) UV–vis–NIR absorption spectra of CuS. (c) Standard equation of CuS solution in the concentration range of 0.1–1 mg/mL. (d) Temperature change with CuS concentrations under infrared light irradiation at 980 nm and a power of 1.0 W/cm^2^. (e) Temperature change over time of CuS solutions (0.1 mg/mL) with different irradiation powers (0–1 W/cm^2^) at 980 nm. (f) Continuous heating and cooling curve of a CuS solution (1 W/cm^2^).

TEM and dynamic light scattering (DLS) results showed that the particle size of the CuS NPs prepared in this study was about 15 nm (Figure 1a). The UV-NIR absorption curve (Figure 1b) demonstrated that the maximum absorption wavelength was 980 nm for the prepared CuS NPs, which is consistent with the previous report.^53^ Therefore, the prepared CuS NPs can be used for photothermal-ablation therapy. Figure 1c shows the standard curve of absorbance for the CuS NP aqueous solution. Linear regression was performed on the absorbance value and the CuS NP concentration, and the linear equation obtained was: *A* = 0.0072*C* – 0.0143 (*R*^2^ = 0.9997), where *A* represents absorbance; *C* represents the concentration of CuS in μg/mL; and *R* represents the fitting correlation coefficient. The temperature of the CuS NP suspension increased rapidly under the irradiation of 980 nm NIR light (1.0 W/cm^2^), and the higher the concentration, the faster the temperature raised (Figure 1d) with 0.1, 0.2, and 0.3 mg/mL of CuS solutions reaching the killing temperature of cancer cells (>50 °C) within 300 s of irradiation. To avoid damage to normal tissues, 0.1 mg/mL was chosen as the optimal concentration for this study. The power of the NIR light had a significant effect on the photothermal effect of the CuS NPs (Figure 1e). Under the NIR irradiation at 1.0 W/cm^2^, the temperature of the CuS NP solution reached 53.5 °C within 300 s, and therefore, it was chosen as the optimal irradiation power. The stability of the photothermal effect of CuS NPs was investigated by applying five heating–cooling cycles (Figure 1f). The CuS NPs exhibited a repeated rise and fall in temperature during each irradiation process. The photothermal effect of CuS NPs is realized via the *d–d* energy band transition of Cu^2+^, which can provide stable and sustainable energy conversion under repeated NIR light irradiation even in a complex *in vivo* environment.

### 3.3 Characterization of Fe_3_O_4_ Nanoparticles

The morphology of the Fe_3_O_4_ NPs was observed using a TEM and a scanning electron microscope (SEM). The prepared magnetic NPs (MNPs) were spherical with a uniform size of 12 ± 3 nm (Figure S2a). The characteristic peaks of the X-ray diffraction (XRD) pattern of the prepared MNPs were consistent with the Fe_3_O_4_ standard diffraction card (19–0629) (Figure S2b). Taking characteristic peaks (220), (310), (400), (422), (511), and (440) into the Debye–Scherrer formula, the average particle size of MNPs was calculated as 12 nm, consistent with the TEM results. The MNPs showed a large infrared absorption peak at 585 nm (Figure S2c), which was consistent with the literature.^54^Figure S2d shows the magnetization results of Fe_3_O_4_. The saturation magnetic strength of the MNPs was 76 emu/g, indicating that they are superparamagnetic materials.

### 3.4 Preparation of Gelatin NPs

#### 3.4.1 Effect of Modified Gelatin Concentration on the Size and PDI of Gelatin NPs

As shown in Table 1, the gelatin concentration had a significant effect on the size and PDI of the final gelatin NPs. The NP size and PDI were, respectively, 116 nm and 0.15 at 1% gelatin concentration and 85 nm and 0.09 at 2% gelatin concentration. The NPs became slightly bigger at higher concentrations of gelatin. Therefore, 2% was selected as the optimal gelatin concentration in this study.

**Table 1:** Effects of Concentration, pH, Antisolvent, Flow Rate Ratio, and Total Flow Rate on Gelatin Nanoparticles

	parameters	mean size (nm)	PDI
effects of gelatin concentration	1%	116	0.15
	2%	85	0.09
	3%	108	0.06
	5%	102	0.07
effects of pH	5.8	1009	0.46
	11	85	0.07
effects of solvent	EtOH	138	0.25
	95% EtOH	103	0.23
	2% Tween-20 in 95% EtOH	85	0.05
effects of flow rate ratio (FRR) (A/S)	1:1	112	1
	1:5	187	0.07
	1:10	205	0.06
	1:20	117	0.06
	1:30	215	0.42
effects of total flow rate (TFR) mL/min	1.05	112	0.11
	10.5	85	0.09
	21	90	0.09
	31.5	90	0.07
	42	97	0.05

#### 3.4.2 Effect of pH on Modified Gelatin NPs

The isoelectric point of gelatin type B is between 4.8 and 5.0,^55^ and within this range, gelatin exhibits low viscosity and low swelling rate, which leads to cross-linking between different gelatin NPs.^55^ Therefore, in this study, a suitable pH was selected to ensure that the surface of gelatin NPs has an appropriate amount of charge so that the NPs are repulsive towards each other, which helps in maintaining good stability. In accordance with previous work,^51^ the pH values of 5.8 and 11 were selected, respectively. As shown in Table 1, when pH was 5.8, the particle size of the prepared NPs exceeded 1000 nm, and their PDI was greater than 0.4, whereas the size of the NPs prepared at pH 11 was less than 100 nm in size, and their PDI was less than 0.1. Therefore, pH 11 was chosen as the optimal pH value in this study.

#### 3.4.3 Effect of the Antisolvent

The antisolvent also showed different effects on NP formation. Tween, as a nonionic surfactant commonly used in drug delivery systems, can effectively reduce the size of NPs.^56^ The principle of its action is the reduction of mutual cohesion between gelatin particles, thus maintaining the stability of the gelatin NP solution and preventing large-scale flocculation. In addition, a reasonable amount of Tween can compete for water molecules against gelatin in the solution so that the water-deficient edge of the gelatin chain tends to shrink at both ends and agglomerate into NPs.^57^ As shown in Table 1, the particle size of the gelatin NPs prepared by using the 95% ethanol solution containing 2% Tween-20 as the organic phase was much smaller (at 90 nm with 0.05 PDI) than that of the gelatin NPs prepared by absolute ethanol and 95% ethanol. Therefore, in this study, 95% ethanol solution containing 2% Tween-20 was selected as the organic phase for the preparation of modified gelatin NPs.

#### 3.4.4 Effects of the Two-Phase Flow Rate Ratio (FRR) and Total Flow Rate (TFR)

The FRR and TFR of the aqueous phase and organic phase had significant effects on the particle size and PDI of NPs (Table 1). When the FRR (A/S) was 1:20, the particle size and PDI of the prepared gelatin NPs were relatively smaller. Although the particle size was the smallest when the FRR (A/S) was 1:1, the PDI of the prepared gelatin NPs turned out to be 1. Therefore, the optimal FRR of the aqueous phase to the organic phase was chosen to be 1:20 in this study. When the FRR was 1:20 and the TFR was greater than 10.5 mL/min, the size of the prepared gelatin NPs was relatively smaller (∼90 nm) and the PDI was less than 0.1. The TFR of 10.5 mL/min was selected for the preparation of gelatin NPs with uniform size and good polydispersity for the following study.

The particle size and preparation time were compared with conventional microsphere preparation methods, such as the water-in-oil emulsion (W/O) method and the emulsion cross-linking method (Table 2). It can be seen from the table that in addition to the preparation using microfluidics, other conventional preparation methods are insufficient to achieve nanoscale gelatin particles, and most of the prepared particles are in the range of 10–50 μm, which are not suitable for use as nanocarriers for drug delivery. It is worth mentioning that the swirl mixer used in this study can prepare gelatin NPs with sizes less than 200 nm within 30 min (Table 1), which has not been reported yet. The preparation time (0.5 h) also includes the curing time of gelatin NPs, as the microfluidic fabrication time only takes a few minutes. Therefore, considering both the size of the NPs and the preparation time, the swirl mixer microfluidic technology has unique advantages and is more suitable for large-scale continuous industrial production.

**Table 2:** Comparison of Different Preparation Methods of Gelatin Particles

preparation methods	size	preparation time (h)	refs
microfluidic method (swirl mixer)	180 ± 10 nm	<0.5	this work
microfluidic method (tapering capillaries)	between tens of microns and hundreds of microns		(58)
emulsion method	5 μm	>6	(59)
emulsion method	12 ± 3 μm	0.75	(60)
water-in-oil emulsion method	13 ± 3 μm	49	(61)
water-in-oil emulsion method	16 ± 4 μm	48	(62)
water-in-oil emulsion method	25 μm	0.75	(63)
water-in-oil emulsion method	40–50 μm	22	(64)
water-in-oil emulsion method	50 ± 18 μm	49	(60, 65)
oil-in-water-in-oil emulsion method	5–40 μm	>8	(65, 66)
emulsion cross-linking method	613 nm	3	(67)
electrohydrodynamic atomization	0.5 μm	>1	(59, 68)

### 3.5 Characterization of Drug-Loaded Gelatin Nanoparticles

Particle sizes, PDI, ζ-potentials, encapsulation efficiency (EE), and loading capacity (LC) of curcumin, CuS, and Fe_3_O_4_ were measured as shown in Figures S3–S6. The entrapment efficiency varied (between 30 and 80%) with the different Cur concentrations studied (1–5 mg/mL). The highest entrapment efficiency was obtained at the Cur concentration of 2 mg/mL (Figure S3). The loading efficiency and particle size increased with the increase in studied Cur concentrations, and the loading efficiency plateaued at 25%, while the ζ-potential decreased (to −20 mV) with the increasing Cur concentration, indicating the increased drug encapsulation. At the highest entrapment efficiency, the size of the nanoparticle was 120 nm with a narrow size distribution (PDI <0.2). When the concentration of CuS was 0.125 mg/mL, the entrapment efficiency was the highest (75%) (Figure S4) with the loading efficiency of around 2%. The particle size increased with the increase in studied CuS concentrations, with the size (200 nm) distribution (0.2) being the narrowest at the concentration of 0.125 mg/mL. When the concentration of Fe_3_O_4_ was 2 mg/mL, the encapsulation efficiency was the highest (77%), while the change of loading efficiency (∼1.3%) was very small (Figure S5). The particle size increased with the increase in Fe_3_O_4_ concentration, and PDI was relatively small when the concentration was 2 mg/mL. The concentration of Fe_3_O_4_ had an insignificant effect on the ζ-potential. The final ratios between Cur, CuS, and Fe_3_O_4_ in the nanoparticles were kept at 25:2:1.3.

**Figure 2:**
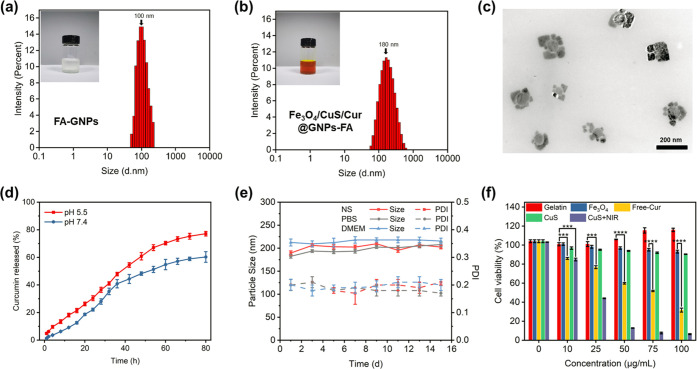
(a) Size distribution of gelatin nanoparticles functionalized with folic acid. (b) Size distribution of Fe_3_O_4_/CuS/Cur@GNPs-FA. (c) TEM of Fe_3_O_4_/CuS/Cur@GNPs-FA. (d) Drug release profiles of Fe_3_O_4_/CuS/Cur@GNPs-FA in PBS at pH 7.4 and 5.5 for 80 h. (e) Stability of drug-loaded gelatin nanoparticles in NS (0.9% NaCl), PBS, and DMEM at pH 7.4. (f) Cytotoxicity of blank gelatin nanoparticles, Fe_3_O_4_, free curcumin, CuS nanoparticles, and CuS + NIR on HFF-1 cells.

The particle size of gelatin NPs functionalized with folic acid (FA-GNPs) was about 100 nm (Figure 2a). Encapsulation of Cur into the FA-GNPs increased the NP size to 120 ± 10 nm (Figure S3c). As shown in Figures S4c and S6, the size of gelatin NPs loaded with CuS NPs (CuS@GNPs-FA) was in the range of 160–240 nm, and the overall solution was pale green due to the presence of CuS NPs. The solution of gelatin NPs loaded with Fe_3_O_4_ NPs and CuS NPs (Fe_3_O_4_/CuS@GNPs-FA) showed an overall brown color, and the particle size increased further (Figure S6). The gelatin NPs loaded with curcumin, Fe_3_O_4_, and CuS NPs (Cur/Fe_3_O_4_/CuS@GNPs-FA) had a darker overall color and a yellowish-brown solution color in the presence of curcumin, and the size was within the range of 180 ± 15 nm (Figure 2b). The morphology of Fe_3_O_4_/CuS/Cur@GNPs-FA, as examined by TEM, is shown in Figure 2c. The particles had a narrow size distribution, which was consistent with the results obtained by DLS.

### 3.6 *In Vitro* Release and Stability of the Drug-Loaded Gelatin NPs

Figure 2d shows the release profiles of Fe_3_O_4_/CuS/Cur@GNPs-FA in PBS containing 20% EtOH and Tween-80 at pH 7.4 and pH 5.5. The addition of ethanol and Tween-80 increased the solubility of hydrophobic drugs.^38^ As can be seen from the figure, the pH of the release medium affects the rate of curcumin release from gelatin NPs. When the pH was 7.4, the gelatin NPs released curcumin slowly. It took 40 h at pH 5.5 and 58 h at pH 7.4 for the release of curcumin from Fe_3_O_4_/CuS/Cur@GNPs-FA to reach 50%. It is known from the literature^62,69^ that under acidic conditions, the gelatin structure relaxes, resulting in changes in the secondary structure of the protein, as a result of which the drug release of Fe_3_O_4_/CuS/Cur@GNPs-FA was promoted at pH 5.5.

To assess the stability of gelatin NPs, the particle size and PDI were measured in PBS (pH 7.4), NS (0.9% NaCl), and DMEM at 4 °C for 15 days (Figure 2e). There were no significant changes in particle size (200 nm), whereas a small fluctuation of PDI (predominantly around 0.2) was observed over 15 days, indicating that gelatin NPs were stable at the physiological condition and thus potent for the delivery of antitumor drugs.

### 3.7 Targeting Effect of the Folic Acid-Modified Gelatin NPs

**Figure 3:**
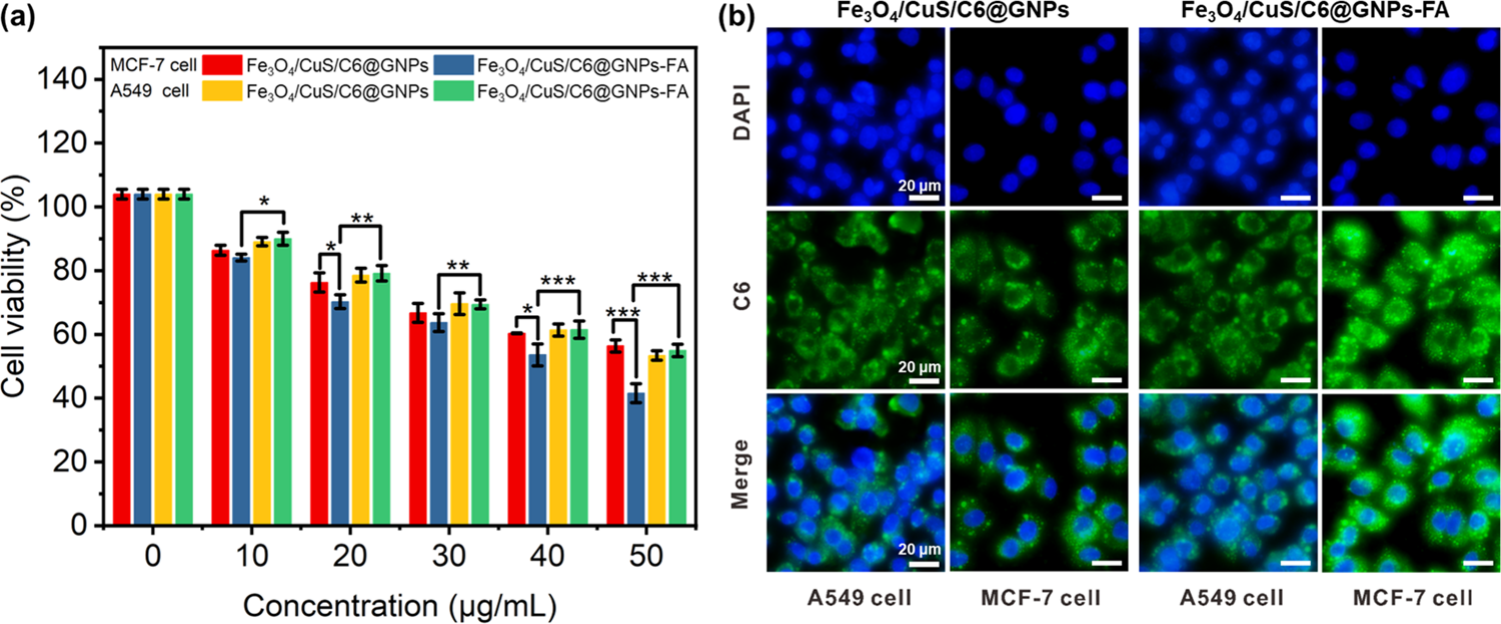
Co-culture of MCF-7 cells and A549 cells demonstrated the targeting effect of folic acid. Cell viability (a) and cellular uptake (b) of MCF-7 and A549 cells incubated with the nonfunctionalized and FA-functionalized GNPs.

MCF-7 cells and A549 cells were co-cultured to test the targeting effect of folic acid (Figure 3a). In order to better observe the uptake effect of different cells on gelatin nanoparticles, coumarin C6 with green fluorescence was used instead of curcumin. According to the literature, folate-grafted nanoparticles were easier to enter MCF-7 cells than A549 cells.^70^ Both Fe_3_O_4_/CuS/Cur@GNPs and Fe_3_O_4_/CuS/Cur@GNPs-FA showed a concentration-dependent inhibition effect on MCF-7 and A549 cells. For MCF-7 cells at the same concentration, the cell inhibition rate of the Fe_3_O_4_/CuS/Cur@GNPs-FA group was higher than that of Fe_3_O_4_/CuS/Cur@GNPs. For A549 cells, the cell inhibition effect of the two drug-loaded gelatin NPs was similar. This indicates that Fe_3_O_4_/CuS/Cur@GNPs-FA has a clear folic acid targeting effect. Figure 3b shows the results of drug uptake on the co-culture of MCF-7 and A549 cells. The fluorescence intensity of MCF-7 cells in the Fe_3_O_4_/CuS/C6@GNPs-FA group was the brightest, while that of other groups was similar, indicating that folate-functionalized gelatin NPs can accelerate the drug intake and accumulation in folate receptor-positive cells through folate receptor-mediated endocytosis, while A549, a folate receptor-negative cell, has no such function. This result indicates that the folate-modified targeted gelatin NPs have a significantly stronger ability to induce apoptosis than nontargeted gelatin NPs.

### 3.8 *In Vitro* Cellular Uptake

**Figure 4:**
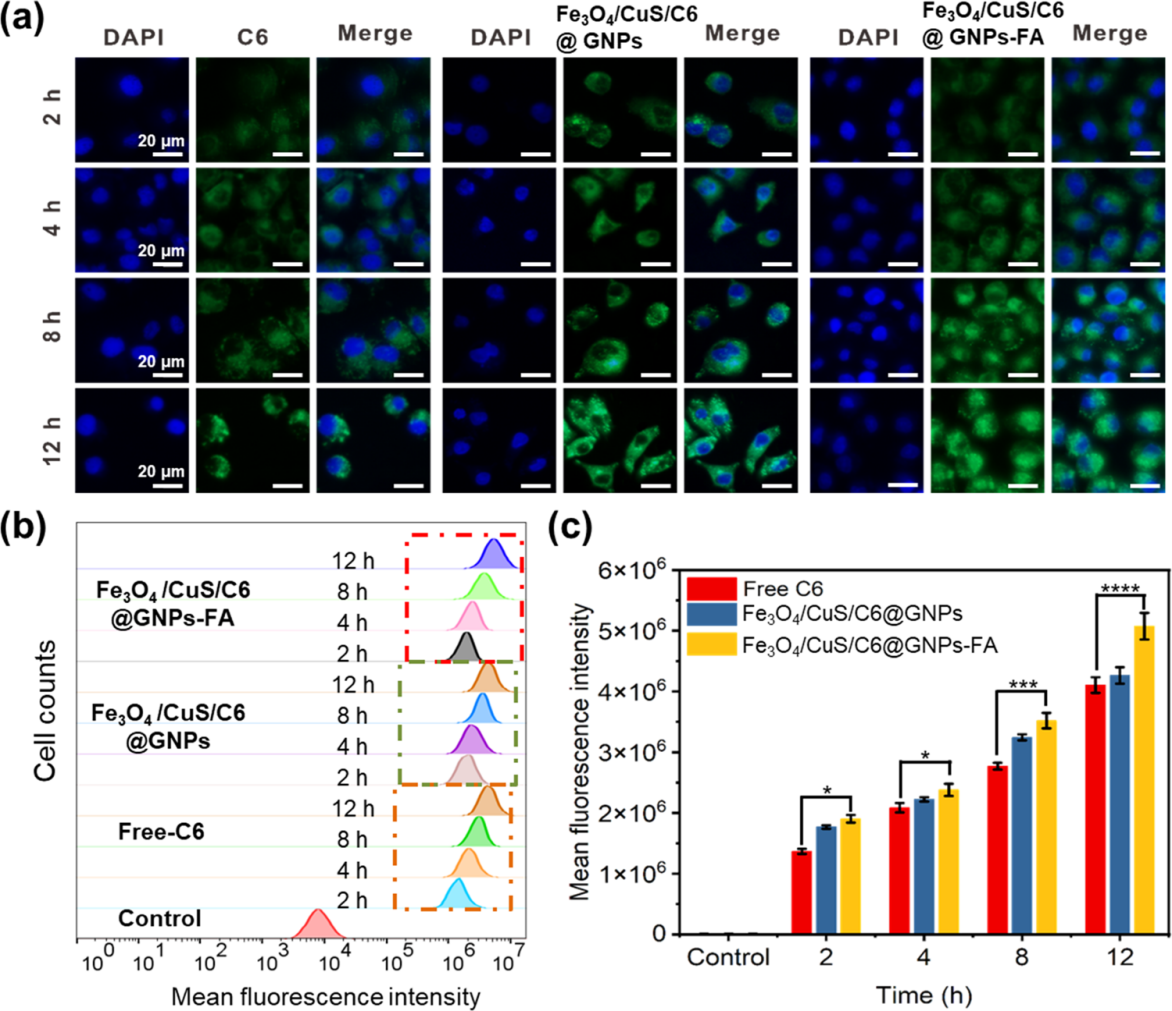
(a) Cellular uptake of NPs by MCF-7 cells. (b) FACS data of C6 fluorescence intensity. (c) Quantitative analysis of drug retention as measured with FACS. The final C6 concentration was controlled at 1 μg/mL.

The qualitative results of the NP uptake by MCF-7 cells, as observed under an inverted wide-field fluorescence microscope, are shown in Figure 4. The time of NP administration also had a significant effect on the NP uptake by MCF-7 cells. The fluorescence intensity of MCF-7 cells treated with Fe_3_O_4_/CuS/C6@GNPs-FA increased as the incubation time increased from 1 to 12 h, indicating that the cellular uptake of gelatin NPs was time-dependent. During the same post-administration incubation time, a stronger green fluorescence signal could be observed in the MCF-7 cells of the Fe_3_O_4_/CuS/C6@GNPs-FA group when compared against the coumarin C6 group and the Fe_3_O_4_/CuS/C6@GNPs group. This is because the surface of MCF-7 cells contains folate receptors, allowing the Fe_3_O_4_/CuS/C6@GNPs-FA to quickly enter cells through the folate receptor-mediated endocytosis. Coumarin C6 is a small fat-soluble molecule, almost insoluble in water, can only enter cells by passive diffusion and also gets easily excreted out of the cells, whereas gelatin NPs enter cells mainly through endocytosis.^71^

**Figure 5:**
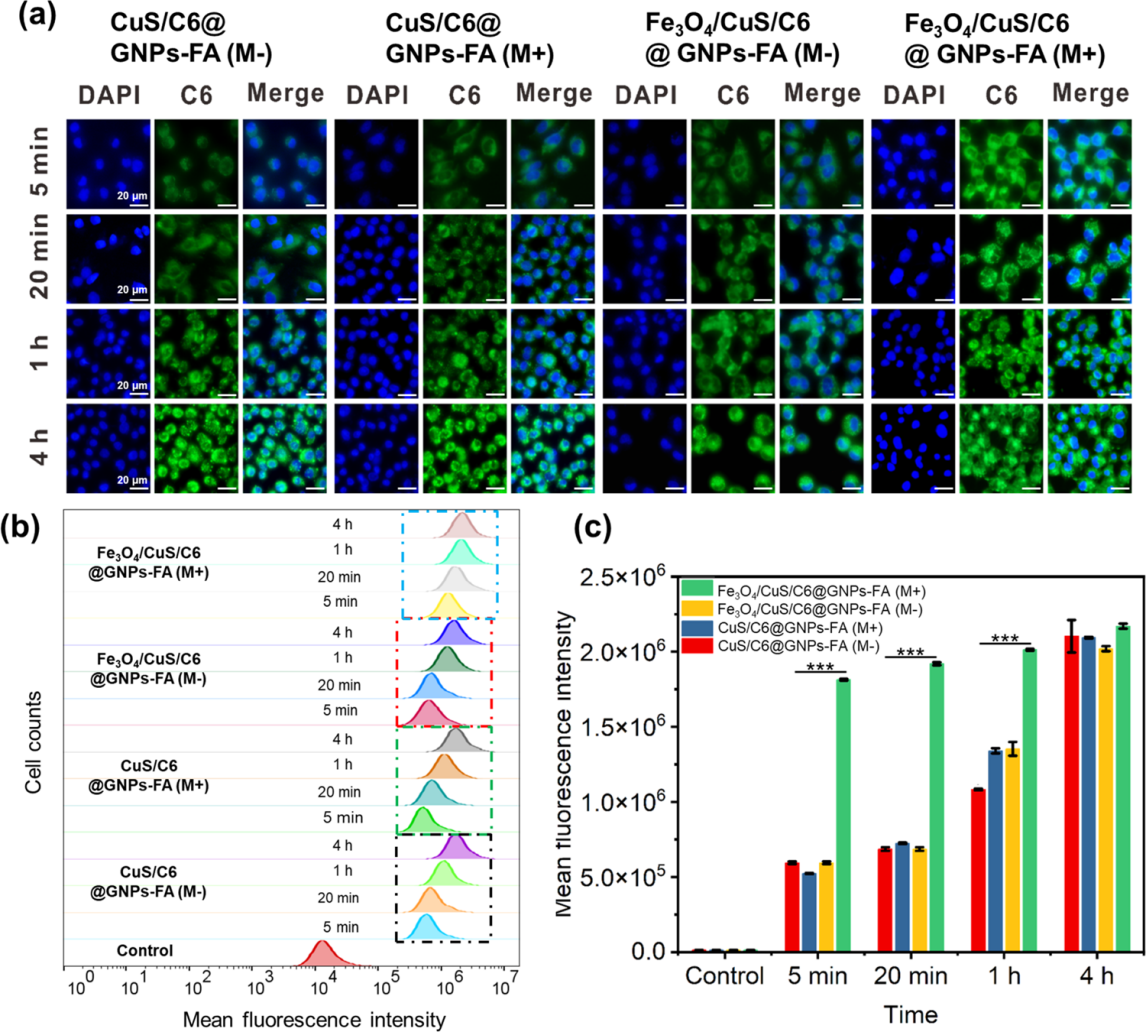
Comparison of the effect of absence and presence of a guiding external magnetic field on (a) GNP uptake by MCF-7 cells; (b) C6 fluorescence intensity as observed in FACS data; and (c) drug retention as measured by quantitative analysis of FACS data. The final C6 concentration was controlled at 1 μg/mL.

Further, flow cytometry was used to quantify the differences in cellular uptake of free coumarin C6, Fe_3_O_4_/CuS/C6@GNPs, and Fe_3_O_4_/CuS/C6@GNPs-FA (Figure 4b,c). The relative fluorescence values showed that the cellular uptake of Fe3O4/CuS/C6@GNPs-FA was significantly higher than that of free Coumarin C6, which was consistent with the results of fluorescence microscopy. CuS/C6@GNPs-FA and Fe_3_O_4_/CuS/C6@GNPs-FA with the applied external magnetic field (CuS/C6@GNPs(M+), Fe_3_O_4_/CuS/C6@GNPs-FA(M+)) and without the external magnetic field (CuS/C6@GNPs-FA(M−), Fe_3_O_4_/CuS/C6@GNPs-FA(M−)) were co-cultured with MCF-7 cells for 5 min, 20 min, 1 h, and 4 h, respectively. The results of the MCF-7 cellular uptake, as observed under an inverted confocal fluorescence microscope, are shown in Figure 5a. The fluorescence intensity of the four groups of MCF-7 cells increased with the incubation time, indicating that the cellular uptake of gelatin NPs was time-dependent. Within the same postadministration incubation time, the CuS/C6@GNPs(M+), CuS/C6@GNPs(M−), and Fe_3_O_4_/CuS/C6@GNPs-FA(M−) groups exhibited similar green fluorescence intensities in MCF-7 cells at 5 min, 20 min, and 1 h. The Fe_3_O_4_/CuS/C6@GNPs-FA(M+) group exhibited significantly stronger fluorescence intensity than the first three groups. At 4 h, more intense green fluorescence signals could be observed in the four groups of MCF-7 cells with little difference in intensity. The above results indicate that Fe_3_O_4_ can be guided by an external magnetic field to target gelatin NPs and accelerate the uptake of drugs by MCF-7, and reduce the treatment time of tumors. Flow cytometry was further used to quantify the difference in cellular uptake (Figure 5b,c). The relative fluorescence values indicated that the cellular uptake of Fe_3_O_4_/CuS/C6@GNPs-FA(M+) was faster than that of other groups, which was consistent with the fluorescence microscopy results.

### 3.9 Cytotoxicity of Gelatin NPs and Their Constituents

To test the cytocompatibility of materials and drug formulations, the viability of HFF-1 cells was assessed using the MTT assay. As shown in Figure 2f, after the blank gelatin NPs were co-cultured with HFF-1 cells for 24 h, the cell viability still reached 100%. This is because gelatin is a high-temperature hydrolyzed product of animal collagen, which is nontoxic and harmless to cells. There was even a weak cell-growth promotion effect, indicating that the blank gelatin NPs have good biocompatibility and can be used as safe carriers for antitumor drugs. Fe_3_O_4_ and CuS NPs were co-cultured with cells at concentrations of 10–100 μg/mL. After 24 h of culture, the cell survival rates were over 95 and 93%, respectively, indicating that Fe_3_O_4_ and CuS NPs could not induce apoptosis under normal conditions, while CuS NPs had a significant inhibitory effect on HFF-1 cells after NIR irradiation. The free anticancer drug curcumin had a clear inhibitory effect on HFF-1 cells.

**Figure 6:**
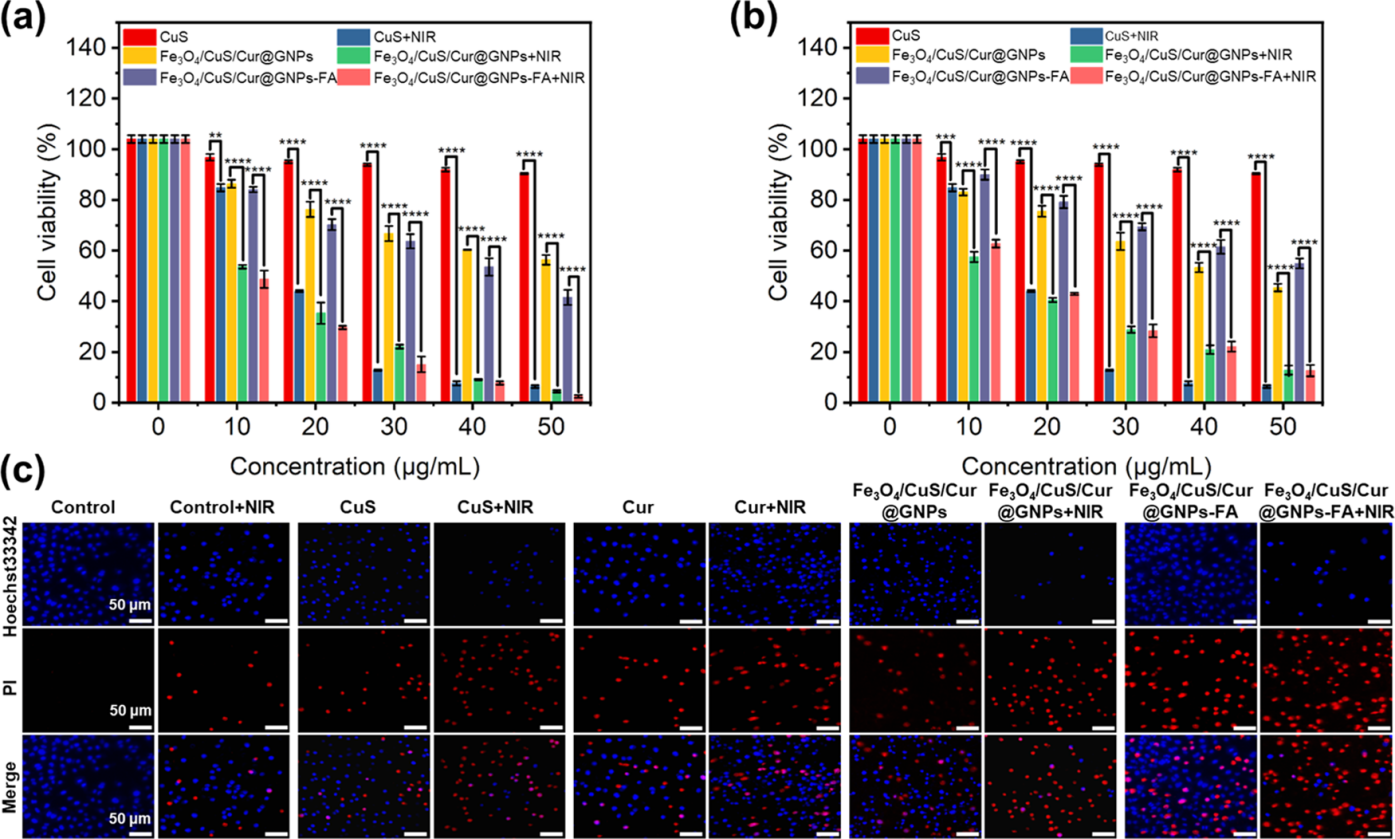
Cytotoxicity of different concentrations of pharmaceutical formulations to MCF-7 cells (a) and A549 cells (b) under NIR light. (*n* ≥ 3;NIR,1.0 W/cm^2^; ****P < 0.0001; ***P < 0.001; **P < 0.01; and *P < 0.05). (c) Live/dead staining of MCF-7 cells, where blue- and red-dyed cells represent live and dead cells at 24 h, respectively.

The targeting effect of folic acid was examined by using MCF-7 cells and A549 cells. As shown in Figure 6a,b, the inhibitory effects of Fe_3_O_4_/CuS/Cur@GNPs and Fe_3_O_4_/CuS/Cur@GNPs-FA on MCF-7 cells and A549 cells were concentration-dependent. With the increase in concentration, the two groups of drug-loaded NPs had a stronger inhibitory effect on cells. For A549 cells, the cytostatic effects of the two drug-loaded gelatin NPs were comparable. In contrast, the inhibition rate of the Fe_3_O_4_/CuS/Cur@GNPs-FA group on MCF-7 cells was greater than that on A549 cells, indicating that CuS/Cur@GNPs-FA had a clear folic acid-mediated cell-targeting effect.

The effect of NIR irradiation on the cell viability of MCF-7 cells and A549 cells was determined by setting up two experiments with and without NIR irradiation. It can be seen from Figure 6a,b that the inhibition rates of the Fe_3_O_4_/CuS/Cur@GNPs group and the Fe_3_O_4_/CuS/Cur@GNPs-FA group with irradiation on MCF-7 cells and A549 cells were much higher than those in the nonirradiated group. The photothermal heating of irradiated CuS NPs enhanced tumor cell-killing ability. Fe_3_O_4_/CuS/Cur@GNPs-FA+NIR had a higher inhibition rate on MCF-7 cells due to folic acid-mediated targeting and photothermal ablation by irradiated CuS.

CuS NPs, Fe_3_O_4_/CuS/Cur@GNPs, and Fe_3_O_4_/CuS/Cur@GNPs-FA were incubated with MCF-7 cells for 24 h. The cells were irradiated with and without NIR before being stained with Hoechst 33,342 and PI to evaluate the killing of tumor cells by gelatin NPs (Figure 6c). It can be seen from the images that the killing efficiency of the four groups of agents on cells is in this order: Fe_3_O_4_/CuS/Cur@GNPs-FA > Fe_3_O_4_/CuS/Cur@GNPs > Cur > CuS. This indicates that folic acid has a targeting effect on MCF-7 cells, and therefore, the intake of Fe_3_O_4_/CuS/Cur@GNPs-FA by MCF-7 is higher than that of other groups, and thus the killing effect is stronger. After irradiation with near-infrared light, the order of cell-killing effect of each group from strong to weak was: Fe_3_O_4_/CuS/Cur@GNPs-FA > CuS > Fe_3_O_4_/CuS/Cur@GNPs > Cur. The reason behind the killing effect of CuS being stronger than that of Fe_3_O_4_/CuS/Cur@GNPs can be speculated that the layers of gelatin on CuS cause a slow rate of photothermal heating of encapsulated CuS upon irradiation compared to free CuS. The red fluorescence (dead cells) in the same group increased significantly after NIR irradiation, except for curcumin, which had no photothermal effect. The blue fluorescence (live cells) of the Fe_3_O_4_/CuS/Cur@GNPs-FA + NIR group was only around 5%. It can be seen that with folic acid-mediated targeting and NIR irradiation, Fe_3_O_4_/CuS/Cur@GNPs-FA had an enhanced killing effect on MCF-7 cells, which is consistent with the conclusion of the MTT experiment.

## 4 Conclusions

In summary, we have successfully used a new swirl mixer to fabricate CuS, Fe_3_O_4_, and curcumin-encapsulated gelatin NPs in one step. Through the exploration of the total flow rate, the flow rate ratio, gelatin concentration, and the antisolvent, the gelatin NPs with desired properties were successfully prepared under optimal conditions. The size of the prepared gelatin NPs was within 200 nm with a small PDI (<0.1), and the NPs remained stable for over 2 weeks. The NP surface functionalization with folic acid enhanced the cellular uptake of the NPs into the MCF-7 cells. The encapsulation of Fe_3_O_4_ into the gelatin NPs also accelerated the cellular uptake of the NPs with a guiding external magnetic field. The photothermal heating of CuS NPs, together with the anticancer effect of curcumin, synergistically killed the MCF-7 breast cancer cells, with the highest killing efficiency of 95%. The experiments proved that the microfluidic preparation of gelatin NPs can well achieve the targeted delivery of anticancer drugs. It is believed that a microfluidic swirl mixer can be used in the pharmaceutical industry for the large-scale preparation of nanodrug delivery systems and will play a key role in the field of anticancer therapy.

## Supporting Information Available

FIIR and ^1^H NMR spectra data (Figure S1); characterization of Fe_3_O_4_ nanoparticles (Figure S2); curcumin encapsulation data (Figure S3); CuS encapsulation data (Figure S4); Fe_3_O_4_ encapsulation data (Figure S5); size distribution of CuS@GNPs-FA and Fe_3_O_4_/CuS@GNPs-FA (Figure S6) (PDF)
