# Supplementary material for: Microfluidic Formulation of Curcumin-Loaded Multiresponsive Gelatin Nanoparticles for Anticancer Therapy
Source: ACS Biomater Sci Eng. 2023 May 4;9(6):3402–13. doi: 10.1021/acsbiomaterials.3c00318 (PMC10265577; doi:10.1021/acsbiomaterials.3c00318)
Supplement: Supplementary file 1 — ab3c00318_si_001.pdf [file ab3c00318_si_001.pdf]

Supporting Information

Microfluidic formulation of curcumin loaded multi-responsive gelatin nanoparticles for anticancer therapy

Yu Xia[1,4], Ruicheng Xu[1,4], Siyuan Ye[1], Jiaxuan Yan[1], Piyush Kumar[2], Peng Zhang[3], Xiubo Zhao[1,2,*]

[1]School of Pharmacy, Changzhou University, Changzhou 213164, China

[2]Department of Chemical and Biological Engineering, University of Sheffield, Sheffield S1 3JD, UK

[3]School of Materials Science and Engineering, Changzhou University, Changzhou 213164, China

[4]The authors contribute equally to this paper.

*Author for correspondence: E-mail: xiubo.zhao@cczu.edu.cn

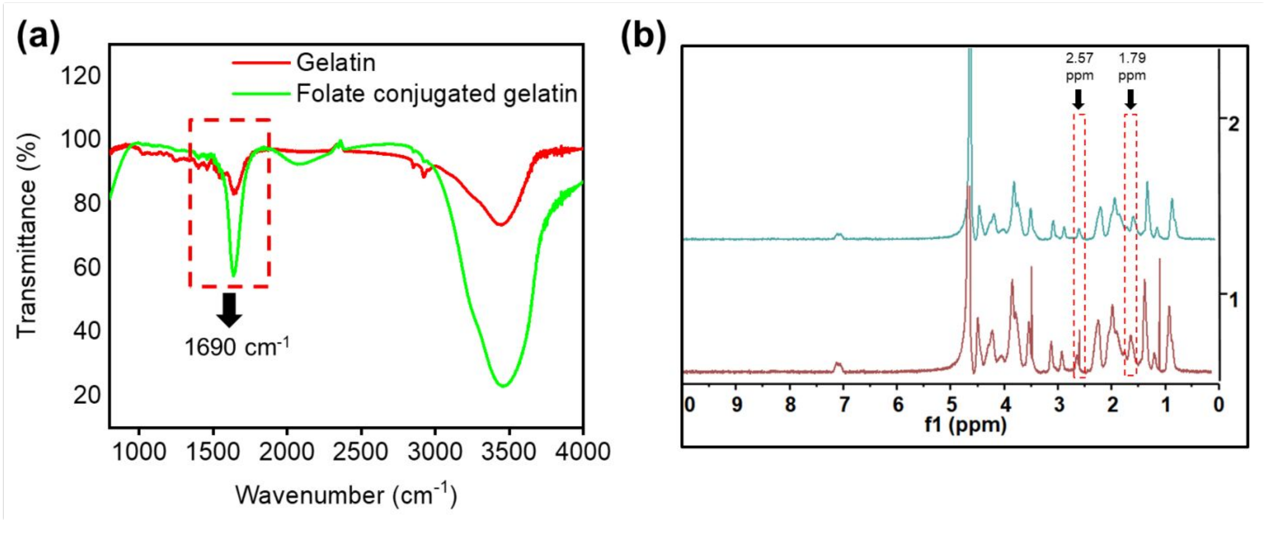

Figure S1. **(a)** FTIR spectra of gelatin (green) and folate conjugated gelatin (red). **(b)** ^{1}H NMR spectra of gelatin (A) and folate conjugated gelatin (B) in D_2O solvent.

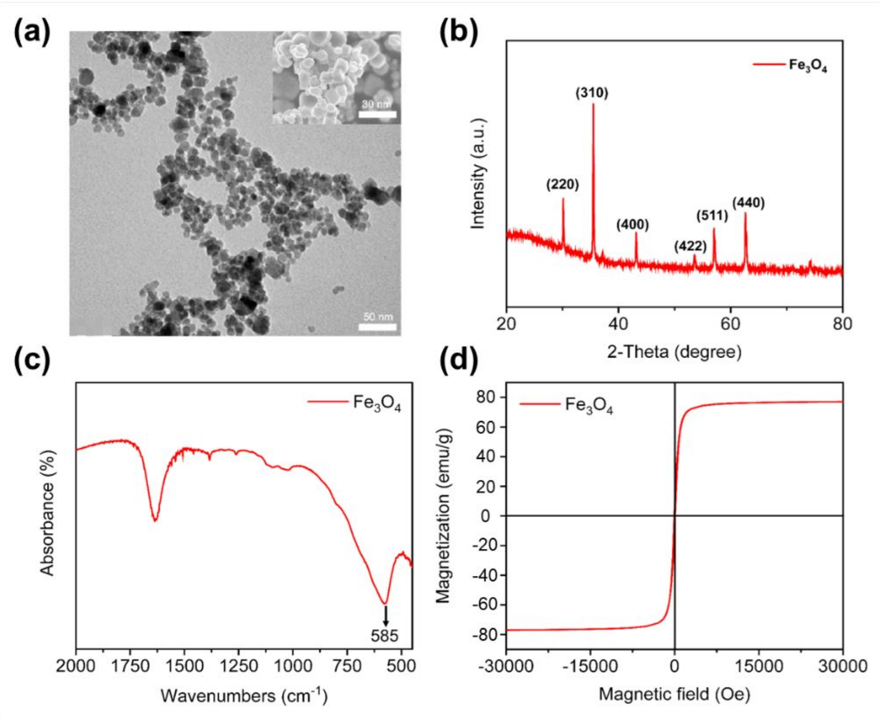

Figure S2. Characterization of Fe_3O_4 nanoparticles. (a) Transmission electron microscopy image of Fe_3O_4 with the SEM image inserted. **(b)** XRD patterns of Fe_3O_4. **(c)** FTIR spectrum of Fe_3O_4. **(d)** Magnetization curve of superparamagnetic Fe_3O_4 NPs.

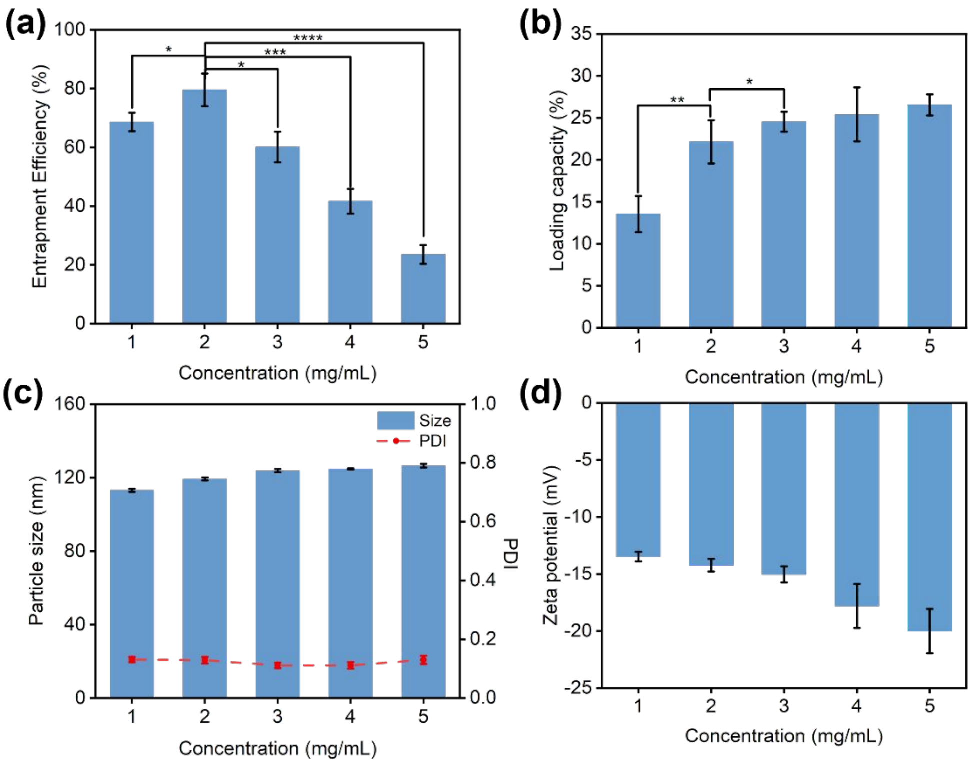

Figure S3. Effect of curcumin concentration on drug entrapment efficiency, particle size, PDI and Zeta potential. **(a)** EE%. **(b)** LC %. **(c)** Particle size and PDI. **(d)** Zeta potential.

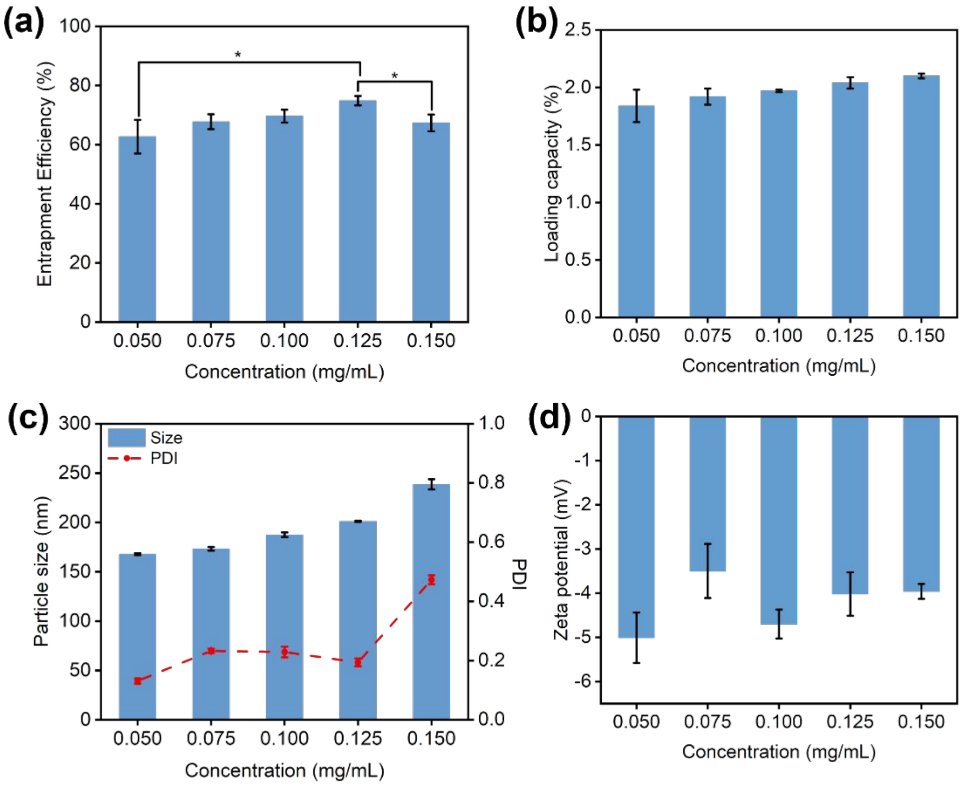

Figure S4. Effect of CuS concentration on CuS entrapment efficiency, particle size, PDI and Zeta potential. **(a)** EE%. **(b)** LC %. **(c)** Particle size and PDI. **(d)** Zeta potential.

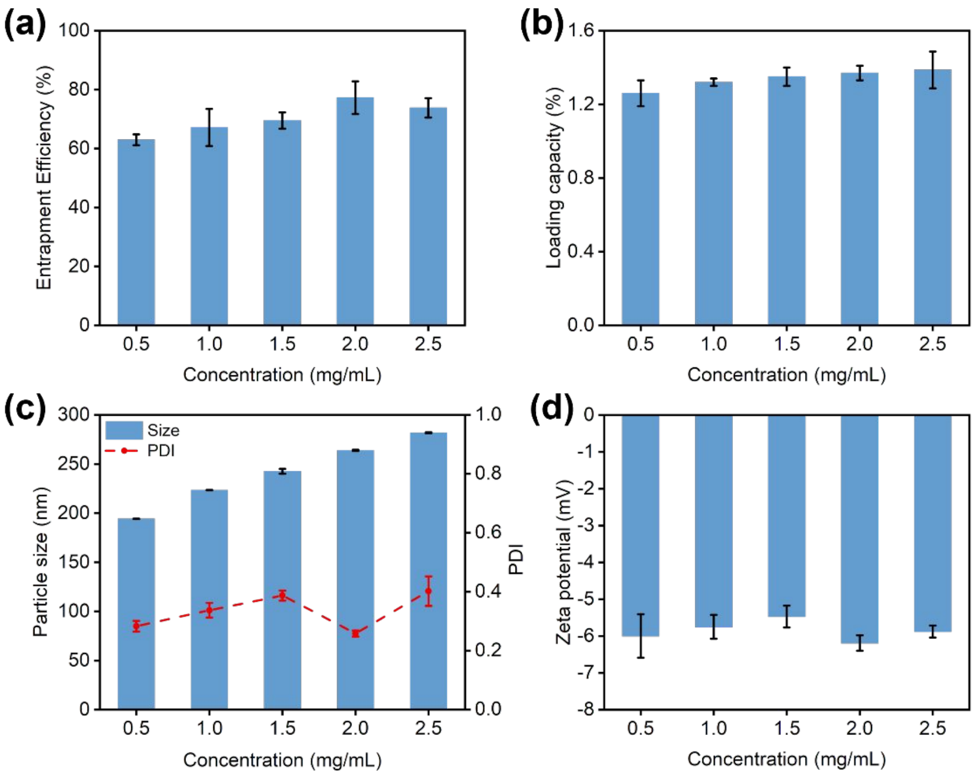

Figure S5. Effect of Fe_3O_4 concentration on Fe_3O_4 entrapment efficiency, particle size, PDI and Zeta potential. **(a)** EE%. **(b)** LC %. **(c)** Particle size and PDI. **(d)** Zeta potential.

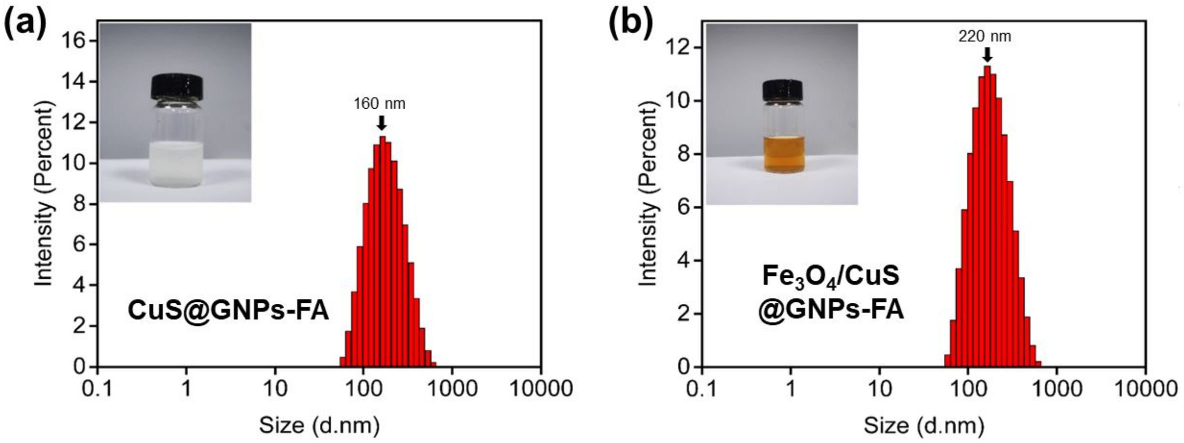

Figure S6. Size distribution of **(a)** CuS@GNPs-FA and **(b)** Fe_3O_4/CuS@GNPs-FA.
